# Herbal Melanin Inhibits Real-Time Cell Proliferation, Downregulates Anti-Apoptotic Proteins and Upregulates Pro-Apoptotic p53 Expression in MDA-MB-231 and HCT-116 Cancer Cell Lines

**DOI:** 10.3390/medicina59122061

**Published:** 2023-11-22

**Authors:** Jothi Ramalingam Rajabathar, Hamad Al-Lohedan, Selvaraj Arokiyaraj, Fathima Mohammed, Dhaifallah M. Al-Dhayan, Norah A. Faqihi, Hassan Al-Saigh

**Affiliations:** 1 Surfactants Research Chair, Chemistry Department, College of Science, King Saud University, P.O. Box 2455, Riyadh 11451, Saudi Arabia; 2 Department of Food Science and Biotechnology, Sejong University, Seoul 05006, Republic of Korea; 3 College of Medicine, King Saud University, Riyadh 11451, Saudi Arabia

**Keywords:** cancer, anticancer agent, herbal melanin

## Abstract

*Background and Objectives*: Cancer is the second-most-important deadly disease in the world, leading to severe socioeconomic consequences and posing a public threat. Consequently, breast and colorectal cancers are significant cancer types that affect women and men more commonly, respectively. Treatment failure or recurrent diseases frequently occur due to resistance, in addition to the side effects of the currently available anticancer agents. Therefore, in this study, herbal melanin anticancer activity was investigated against human breast adenocarcinoma (MDA-MB-231) and human colorectal (HCT 116) cell proliferation and the expression of downregulated anti-apoptotic proteins and upregulated pro-apoptotic p53. *Materials and Methods*: MDA-MB-231 and HCT 116 cells were monitored for their real-time proliferation properties using Xcelligence. Herbal melanin of various concentrations significantly inhibited MDA-MB-231 and HCT 116 cell proliferation. Then, the expression of proapoptotic and anti-apoptotic proteins such as p53, Bcl-2 and Bcl-xl was studied using Western blotting. *Results*: The Bcl-2 and Bcl-xl expressions were downregulated, while the p53 expression was upregulated after treatment with herbal melanin. Similarly, the expression of apoptotic proteins such as Bcl-2, Bcl-xl, XIAP, Survivin, Bid, Bax, p53, Cytochrome C, PARP genes and mRNA was studied after herbal melanin treatment using real-time PCR, which revealed the downregulation of Bcl-2, Bcl-xl, XIAP and Survivin and the upregulation of Bid, Bax, p53, Cytochrome C and PARP apoptotic protein expression. Also, caspase 3 and 9 expressions were monitored after the treatment with herbal melanin, which revealed the upregulation of both the MDA-MB-231 and HCT 116 cell types. *Conclusions*: Overall, herbal melanin can be used as an alternative anticancer agent against the MDA-MB-231 and HCT 116 cell types.

## 1. Introduction 

Cancer constitutes a huge collection of diseases that can affect any part of the body. Consequently, cancer burdens as well as deaths are increasing globally. Cancer is the primary cause of death due to its widely spreading nature. By 2030, nearly 22 million cases are estimated, because each year, several million cases are encountered [1,2]. Cancer is the second-most-common cause of death, creating a serious menace to public health and resulting in a severe socio-economic burden. Most importantly, the incidence or prevalence of cancer is increasing in developed and developing countries due to exposure to various risk factors such as alcohol consumption, poor diet, physical inactivity and air pollution [3,4]. The report says geographical diversity also plays a role in particular cancer types (e.g., cervical cancer), but the worldwide cancer burden has revealed an unexpected increase in malignant types that are associated with modern lifestyles, such as breast, colon and lung cancers [5]. Out of all types of cancer, breast cancer was detected as the leading cancer type in women in terms of causing death, and colorectal cancer is also an important cancer type in men as well as women [5,6,7]. These types of cancer are mainly prevented by avoiding risk factors, early diagnosis, appropriate treatment options and patient care. Fortunately, most cancer types are cured if detected early and treated properly. But the primary reasons for treatment failure for both breast and colorectal cancers are poor diagnosis and inappropriate treatment procedures that are not sufficient for disease control. The appropriate treatment methods involve surgeries followed by chemotherapy, and radiotherapy as well as hormone therapies are recommended for all phases of cancer treatment. Still, treatment failure does occur owing to drug resistance and unwanted side effects caused by the currently available therapeutics, which limits the efficacy of current treatment procedures [8,9]. Therefore, the prevention and control of both breast and colorectal cancers are more critical. Hence, the current situation prompted researchers to find alternative ways for breast and colorectal cancer drug resistance prevention, as well as no or lower side effects. Meanwhile, when chemotherapy is ineffective, there is a crucial need to find new agents against breast and colorectal cancers. 

Numerous studies have declared the anticancer activities of different phytochemicals as well as herbal products against various types of cancer, including breast and colorectal cancer. Therefore, herbal melanin has played a major role in many treatment processes owing to its wide range of biological activities, such as anti-ulcer, anti-inflammatory and anti-oxidant [10,11,12]. Generally, melanin belongs to a dark brown to black multifunctional pigment family responsible for tissue darkness and preventing radiation such as visible light, ultraviolet and heat, and it can be derived from various plants, animals and microbes [13,14]. Most importantly, melanin is generally connected to wide range of immune response in different living things [15,16]. Hence, herbal melanin is known for its various biological properties, including its radical scavenging capacity and antioxidant activity. Recently, herbal melanin was evaluated for its anticancer activity against various cancer cell types [17,18]. Cancer treatment faces significant challenges, including drug resistance and the unwanted side effects associated with conventional therapeutics. The quest for alternative approaches has led to the exploration of herbal melanin, which holds promise for influencing cell proliferation and the regulation of anti-apoptotic proteins. This manuscript aims to provide a comprehensive overview of the potential therapeutic benefits of herbal melanin in the context of cancer treatment. Based on this evidence, in this study, the anticancer properties of herbal melanin were investigated on breast and colorectal cancer cell types, and their effect was studied on the migration and signaling pathways of breast and colorectal cancer cell types. 

## 2. Materials and Methods

### 2.1. Reagents

All the reagents used in the study were purchased from Sigma Aldrich and Invitrogen. The herbal melanin used in the study was extracted previously, and its physical characterizations were reported earlier [19]. Throughout the study, all the experiments were conducted in triplicate.

### 2.2. Cell Culture Preparation

In the study, human breast adenocarcinoma (MDA-MB-231) and human colorectal (HCT 116) cell lines procured from ATCC (American type culture collection, Manassas, VA, USA) were maintained in Dulbecco’s modified Eagle medium (DMEM, Invitrogen, Waltham, MA, USA) supplemented with 10% fetal bovine serum, streptomycin (100 µg/mL), penicillin (100 IU/mL) and L-glutamine (2 mmol/L) and incubated at 37 °C with 5% CO_2_. The cells were passaged every 2–3 days using enzymatic digestion after confluence and split at a ratio of 1:2 or 1:3. The cells were used between passages 5 and 9 for the study. 

### 2.3. Real-Time Cell Proliferation by the xCELLigence System

The effect of HM on MDA-MB-231 and HCT 116 cell proliferation were assessed in real time using the xCELLigence Real-Time Cell Analyzer Dual Plate (RTCA-DP) system according to the manufacturer’s recommendations (Acea, Biosciences Inc., San Diego, CA, USA). Briefly, MDA-MB-231 cells (5 × 10^3^/well) and metastatic HCT 116 cells (12 × 10^3^/well) in 150 µL medium/well were cultured in a 16-well E-plate (ACEA Biosciences Inc., San Diego, CA, USA) for a cell proliferation assay. For the cell proliferation assay, the cell treatment was performed the next day of the cell seeding. The cell index values were monitored every 15 min for 30 h for cell proliferation. For cell proliferation assessment, baseline cell indices were calculated for at least two measurements from three replicate experiments. 

The MTT assay conducted in this study provides a quantitative assessment of cell viability following treatment with herbal melanin (HM) at varying concentrations. The initial step involved seeding cells at a density of 5 × 10^3^ in a 96-well plate, providing a standardized environment for the subsequent experimental conditions. After a 24 h incubation period to allow for cell adherence and acclimatization, the cells were either left untreated (considered as the control) or exposed to HM at concentrations ranging from 5 to 200 μg/mL for an additional 24 h.

The addition of 3-(4,5-dimethylthiazol-2-yl)-2,5-diphenyltetrazolium bromide (MTT) solution to the cells initiated the formation of formazan crystals, directly proportional to the metabolic activity and viability of the cells. The 2 h incubation period allowed for the completion of this reaction. Subsequently, the addition of dimethyl sulfoxide (DMSO) dissolved the formazan crystals, and the absorbance of the resulting product was measured at 540 nm using a Synergy™ 2 multi-mode microplate reader (BioTek Instruments, Inc., Winooski, VT, United States). Performing the experiments in triplicate for each condition enhanced the reliability and statistical robustness of the results, providing a comprehensive understanding of the concentration-dependent effects of HM on cell viability. The choice of these experimental parameters, from cell seeding to the specific concentrations of HM and the duration of incubation, demonstrates a meticulous approach in designing the assay. The use of a multi-mode microplate reader for absorbance measurements added precision to the quantitative analysis.

### 2.4. RNA Extraction and Quantitative Real-Time Reverse Transcription-Polymerase Chain Reaction

Both MDA-MB-231 and HCT-116 cells (1 × 10^6^/dish) were cultured in a 100 mm dish up to 60% confluency in the complete medium for 24 h of incubation. The next day, the cells were treated with 10 μM of HM for a further 24 h of incubation. The total RNA was extracted from the treated and untreated cells using a PARIS™ kit (Ambion Inc., Austin, TX, USA). A High-Capacity cDNA kit (cat. no. 4368814) was used for reverse transcription (Applied Biosystems, Waltham, MA, USA). The RNA quality was evaluated by assessing the A260/280 ratio (1.8–2.0) using a NanoDrop ND-2000 UV-VIS spectrophotometer (Thermo Fisher Scientific, Inc., Waltham, MA, USA). A quantitative PCR analysis was performed on ViiA™ 7 real-time PCR system (Thermo Fisher Scientific) using SYBR Green PCR Master Mix (cat no 4385612, Thermo Fisher Scientific). The relative mRNA expression levels of Bcl-2, Bcl-xl, XIAP, Survivin, Bid, Bax, Cytochrome c, p53, PARP, Caspase 3 and Caspase 9 were normalized to GAPDH for a quantitative real-time reverse transcription-polymerase chain reaction (qRT-PCR). The primer sequences are listed in Table 1. For each gene analysis, a negative control was prepared without a cDNA template. All the reactions were performed in triplicate and repeated in each experiment three times separately.

### 2.5. Western Blotting

Whole cell lysates were prepared using radio immune precipitation assay (RIPA) lysis buffer (Boston Bio products, Ashland, MA, USA) and protein concentration was determined using the Bradford Protein reagent (Bio-Rad laboratories Inc., Hercules, CA, USA). Equal amounts of cell lysate proteins were loaded and electrophoresed using 4–20% Mini-Protean TGX precast gels (Bio-Rad) and subsequently transferred to a 0.22 μm nitrocellulose transfer membrane using the trans-blot turbo transfer system (Bio-Rad). The membranes were blocked in 5% skimmed milk in PBS containing 0.1% Tween-20 (PBST) for 1 h at room temperature and were incubated overnight at 4 °C with the primary antibodies listed in Table 1. Immuno-reactivity occurred after incubation with horseradish peroxidase-conjugated secondary antibodies for chemiluminescence detection using Clarity Western ECL Substrate (Bio-Rad). The images were captured using a C-DiGit™ Blot Scanner (LI-COR Biosciences, Lincoln, NE, USA) and analyzed using Image Studio™ software (LI-COR Biosciences).

### 2.6. Statistical Analysis

The results are expressed as the mean ± standard deviation (SD). Data points were collected for a minimum of three independent experiments. A one-way ANOVA test was used to compare two groups and a value of *p* < 0.05 considered significant. The use of the mean ± standard deviation provides a clear representation of the data distribution. Additionally, conducting a minimum of three independent experiments added robustness to our findings, ensuring reliability. The choice of the one-way ANOVA test for group comparison is appropriate, and setting a significance level of *p* < 0.05 is a standard practice in scientific research. This statistical approach strengthens the validity of our results and contributes to the overall credibility.

## 3. Results

### 3.1. HM Reduced Real-Time Cell Proliferation in Breast Adenocarcinoma and Colorectal Cancer Cell Lines

**Figure 1:**
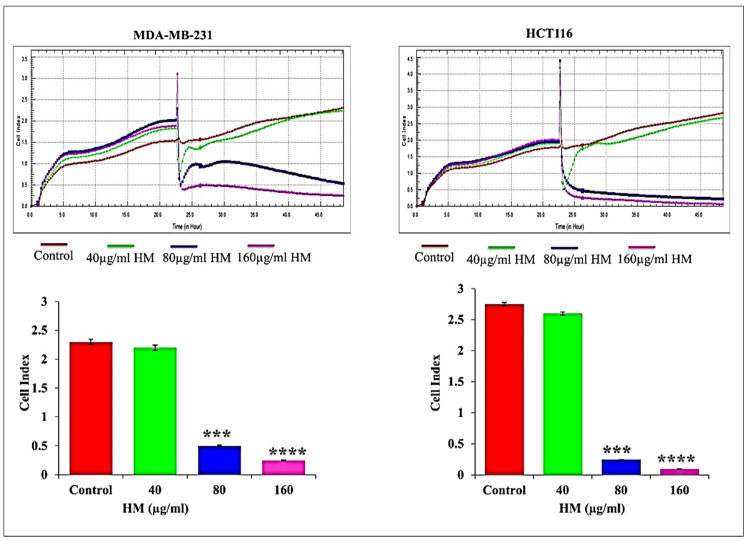
Real-time cell proliferation MDA-MB-231 and HCT116 were monitored using the xCELLigence RTCA-DP system. Bar graphs showing the results expressed as mean ± SD of three independent experiments (*n* = 3). The data were considered significant when reporting *** *p* < 0.001, and **** *p* < 0.0001 vs. control.

The real-time monitoring of herbal melanin’s effect on MDA-MB-231 and HCT-116 cell proliferation was studied and the calculated cell indices after HM treatment are presented in Figure 1. Here, herbal-melanin-treated MDA-MB-231 and HCT-116 cancer cells were subjected to one of the cell-based assays to monitor the proliferation profiles, which are important activity in the development and progression of cancer. The real-time monitoring system functioned based on the measurement of electrical impedance obtained from a record of the cell index in both the cancer cell types.

The cell index represents the dimensional parameter resulting in change in the electrical impedance measurement, which reflects cell physiological conditions such as proliferation. The herbal melanin gradually inhibited the cell proliferation upon increasing the herbal melanin concentrations against both MDA-MB-231 and HCT-116 cell types when compared to untreated cells. Using the cell index as a measure of cellular physiological conditions is a clever way to assess changes in cell proliferation. The observation of the untreated cells reaching the maximum cell index, indicative of confluency, serves as a helpful baseline for comparison. The gradual inhibition of cell proliferation with increasing concentrations of herbal melanin against both MDA-MB-231 and HCT-116 cell types aligns with the idea of herbal melanin influencing cell proliferation, as suggested by the earlier part of the manuscript.

### 3.2. HM Induced Downregulated Anti-Apoptotic and Upregulated Pro-Apoptotic Proteins Expression in Both Cell Lines

**Figure 2:**
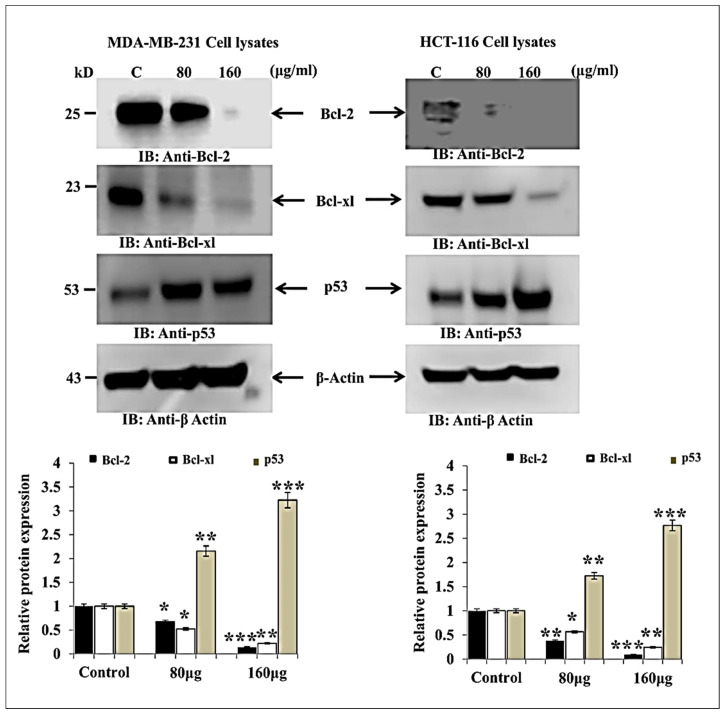
Total MDA-MB-231 and HCT116 cell lysates were immunoblotted (Bcl-2, Bcl-xl, p53). Bar graph indicating the relative protein expression level related to the loading control *ß*-actin (*n* = 3). * *p* < 0.05, ** *p* < 0.01, *** *p* < 0.001 vs. control.

The herbal melanin effect on MDA-MB-231 and HCT-116 cell pro-apoptotic proteins such as p53, Bcl-2 and Bcl-xl were investigated using Western blot and the obtained results are presented in Figure 2. As seen in the figure, the Bcl-2 and Bcl-xl protein expressions were inhibited after treatment with herbal melanin in both MDA-MB-231 and HCT-116 cells when compared to untreated control. Meanwhile, p53 suppressor protein expression was significantly upregulated in both MDA-MB-231 and HCT-116 cells when compared to untreated control. 

### 3.3. HM Induced Downregulated Anti-Apoptotic and Upregulated Pro-Apoptotic mRNA Expression in Both Cell Lines

**Figure 3:**
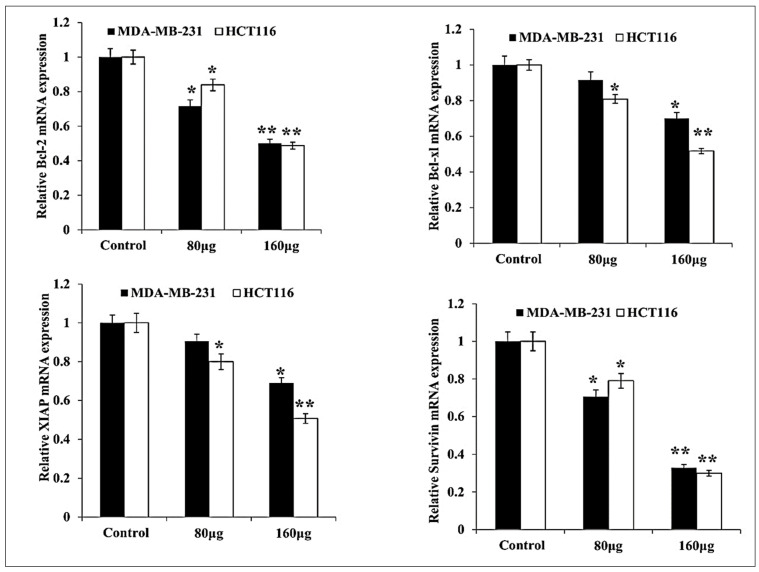
Bar graph showing the relative mRNA expression levels of Bcl-2, Bcl-xl, XIAP and Survivin related to the internal control GAPDH monitored in MDA-MB-231 and HCT116 RNA extracts (*n* = 3). The data were considered significant when reporting * *p* < 0.05, ** *p* < 0.01, vs. control.

**Figure 4:**
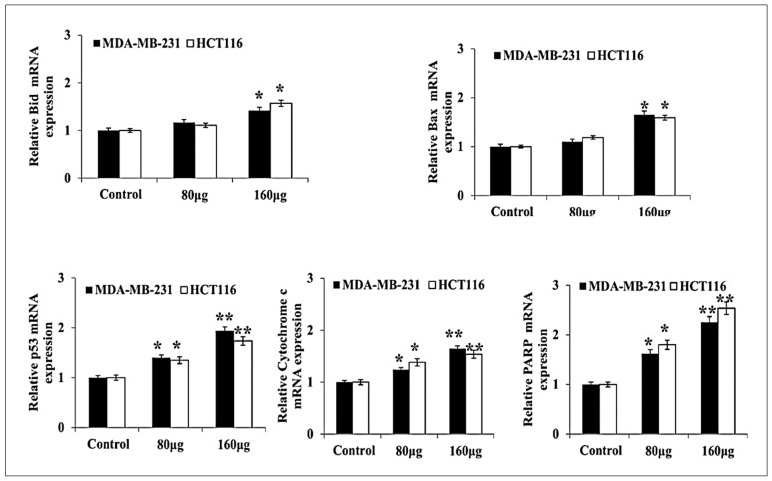
Bar graph showing the relative mRNA expression level of Bid, Bax, p53, Cytochrome c and PARP related to the internal control GAPDH monitored in MDA-MB-231 and HCT116 RNA extracts (*n* = 3). The data were considered significant when reporting * *p* < 0.05, ** *p* < 0.01, vs. control.

The herbal melanin effect on MDA-MB-231 and HCT-116 cell pro-apoptotic and anti- apoptotic proteins such as Bcl-2, Bcl-xl, XIAP, Survivin, Bid, Bax, p53, Cytochrome C and PARP were investigated using real-time PCR and the obtained results are presented in Figure 3 and Figure 4. As shown in the figure, the Bcl-2 expression was down-regulated in both MDA-MB-231 and HCT-116 after treatment with two different concentrations of herbal melanin. In the same way, Bcl-xl expression was reduced in both MDA-MB-231 and HCT-116 after treatment with concentrated herbal melanin. Similarly, the X-linked inhibitor apoptosis protein (XIAP) overexpression was downregulated in both MDA-MB-231 and HCT-116 cell types after treatment with herbal melanin. Similarly, the Survivin overexpression was reduced in both MDA-MB-231 and HCT-116 cell types after treatment with herbal melanin. As presented in Figure 4, the Bid, Bax, p53, Cytochrome C and PARP proteins were upregulated when treated with two different concentrations (80 µg and 160 µg) of herbal melanin. 

### 3.4. HM Induced Upregulated Caspase 3, 9 Expressions in Both Cell Lines

**Figure 5:**
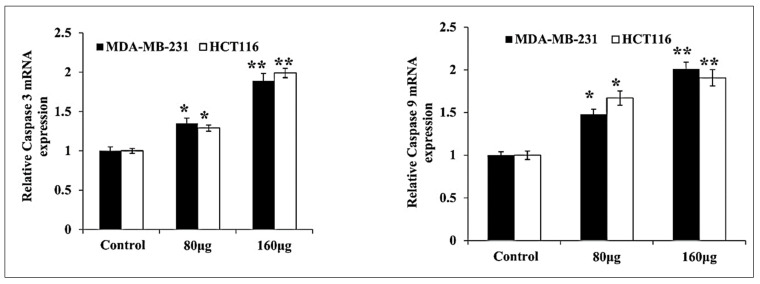
Bar graph showing the relative mRNA expression levels of Caspase 3, Caspase 9 related to the internal control GAPDH monitored in MDA-MB-231 and HCT116 RNA extracts (*n* = 3). The data were considered significant when reporting * *p* < 0.05, ** *p* < 0.01, vs. control.

The herbal melanin effect on MDA-MB-231 and HCT116 cells for caspase 3 and 9 expression was investigated and the obtained results are presented in Figure 5. As mentioned in Figure 5, the up-regulated expression of caspase 3 and 9 was observed in both MDA-MB-231 and HCT116 cells after treatment with various concentrations of herbal melanin. 

## 4. Discussion

Nowadays, cancer is most common and significant painful or shocking disease representing the second primary reason for death [20]. Many successful treatment strategies are available to treat cancer-infected patients; however, treatment failures still occur owing to the development of resistance towards currently available anticancer agents [20,21]. Therefore, there is an urgent need for alternative anticancer agents that fight against breast and colorectal cancer cells, which create a huge threat to public health. Many studies have proved the anticancer activity of various herbal products and their safe nature in treating various cancer types [22]. Keeping these facts in mind, the anticancer properties of herbal melanin were investigated against human breast adenocarcinoma (MDA-MB-231) and human colorectal (HCT 116) cells. Mainly, this novel anti-cancer agent was initially screened for its specific target sites, which are important for controlled rules for cancer cell proliferation, apoptosis, mutated or over-expressed proteins and cell cycle regulation. To confirm the specific target sites of herbal melanin, our study monitored the real-time proliferation potential of MDA-MB-231 and HCT 116 cells after treatment with herbal melanin at different concentration using an xCELLigence Real-Time Cell Analyzer Dual Plate (RTCA-DP) system and proved the activity of herbal melanin on cell proliferation by inhibiting the growth of MDA-MB-231 and HCT 116 cells. This real-time technique was used to monitor the adhesion and proliferation properties of cancer cells. Here, our result was correlated with an earlier report wherein the anticancer activity of crinamine extracted from *Crinum asiaticum*, a persistent herb, was evaluated against cervical cancer and effectively inhibited proliferation and migration, monitored through xCELLigence. This activity was achieved by suppressing the expression of positive regulators of epithelial mesenchymal transition SNAl1 and VIM [23]. In support of this, a study revealed the cytotoxic effect of new benzofuran isatin conjugate anti-cancer activity against colorectal adenocarcinoma HT29 and metastatic colorectal cancer SW620 cell type proliferation, migration and invasion, monitored after treatment using an xCELLigence real-time system, finding that it potentially inhibited colorectal cancer cell proliferation, migration and invasion when compared to untreated cells [24]. Another study investigated the anti-cancer activity of vanadium pentoxide against colorectal (colo-205) and breast cancers (MCF-7) and their proliferation and migration profiles using the xCELL ligence real-time system, and the compound was found to effectively inhibit the proliferation and migration properties of both cell types after treatment [25]. In the same way, the anticancer activity of *N. sativa* was evaluated alone and in combination with the known anticancer agent doxorubicin against breast cancer and exhibited potent activity by inducing cell death via changes in morphology and inhibition in cell proliferation [26]. 

The observed inhibition of cell proliferation with increasing concentrations of herbal melanin against both MDA-MB-231 and HCT-116 cell types is a compelling finding that underscores the potential therapeutic impact of herbal melanin. This concentration-dependent response suggests a dose–response relationship, indicating that herbal melanin may exert its inhibitory effects in a manner correlated with its concentration.

The gradual decrease in cell index implies a progressive reduction in cell proliferation, a critical aspect in cancer treatment. The fact that this effect is consistent across different cell types (MDA-MB-231 and HCT-116) suggests a broad applicability of herbal melanin, making it a promising candidate for a wide range of cancers.

The concentration-dependent trends observed in this study prompt further exploration into the optimal dosage of herbal melanin for maximum efficacy. Fine-tuning the concentration could potentially enhance its therapeutic benefits while minimizing any potential adverse effects.

The comparison with untreated cells serves as a crucial control, providing a baseline for evaluating the relative impact of herbal melanin. The significant differences observed, as indicated by the one-way ANOVA test, emphasize the specific and targeted nature of herbal melanin in inhibiting cell proliferation.

While these findings are promising, it is essential to consider potential mechanisms underlying the observed effects. Further studies could delve into the molecular pathways influenced by herbal melanin, shedding light on its mode of action and potentially uncovering new targets for cancer intervention.

Moreover, the translation of these in vitro results to in vivo models and eventually clinical trials is a critical next step. Understanding the effectiveness and safety profile of herbal melanin in a more complex biological context will be pivotal in establishing its viability as a potential cancer therapeutic.

In conclusion, the concentration-dependent inhibition of cell proliferation by herbal melanin against MDA-MB-231 and HCT-116 cell types presents a compelling case for its therapeutic potential. These findings lay the groundwork for future investigations into the underlying mechanisms, optimal dosage, and translational potential of herbal melanin in the realm of cancer treatment.

Besides cell proliferation, herbal melanin was investigated for its anti-apoptotic activity. More importantly, programmed cell death or apoptosis is an important process activated by DNA damaging agents or the use of chemotherapeutic agents. Apoptosis is the main regulator of physiological growth control as well as tissue homeostasis. Apoptosis is critically involved in the formation of tumors and also controls the treatment response [27]. Apoptosis has an association with resistance increases to anti-cancer agents, thereby limited future possibility for apoptosis. Hence, apoptosis is an important target for novel anticancer agents. Apoptosis is mainly mediated by several proteins such as p53, BCl-2 and BCL-xl and their genes. Therefore, our study investigated the expression of apoptotic proteins such as p53, BCl-2 and BCL-xl after treatment with herbal melanin using Western blotting. Here, p53 is a tumor suppressor factor that regulates cell functions such as apoptosis promotion and senescence and is also involved in the suppression of cell growth, invasion and migration [28]. During apoptotic stimulation, p53 shifted from the cell nucleus to mitochondria and interacted with Bcl-2 family proteins to regulate apoptosis. This family consists of prosurvival (Bcl-2, Bcl-w and Bcl-xl), multidomain proapoptotic (Bax and Bak) and BH3-only (Bid and Bad) groups. In healthy cells, p53 regulates apoptosis through the mitochondrial pathway of apoptosis. The Bcl-2 protein controls cell migration and invasion, resulting in cancer metastasis. This process is encouraged by Bcl-2, Bcl-XL and Bcl-w and suppressed by the multidomain proapoptotic group. p53 is activated by the control of Bcl-2 family genes. The Bcl 2 family includes more important mediators for mitochondrial outer membrane permeabilization involved in apoptosis [29] and also plays a major role in cytochrome C release from mitochondria [30]. Overexpression of Bcl-2 inhibits cell death via many factors including hypoxia, oxidative stress and growth factor deprivation. Therefore, our study reported inhibition in Bcl-2 and Bcl-Xl expression and that p53 expression was upregulated after treatment with herbal melanin. Our study was correlated with an earlier report that investigated tea tree oil extract for anticancer activity against skin cancer cell line A-375 and Hep-2 cell lines and studied the apoptotic regulatory genes p53, BAX and BCL-2 using real-time PCR and Western blot. In addition, tea tree oil activated caspase 37 and 9, p53 upregulation and the downregulation of anti-apoptotic genes [31]. 

In addition, the pro-apoptotic and anti-apoptotic protein genes such as Bcl-2, Bcl-xl, XIAP, Survivin, Bid, p53, Cytochrome C, PARP mainly involved in apoptosis were analyzed in MDA-MB-231 and HCT-116 cells after treatment with herbal melanin. Here, Bcl-2, Bcl-xl, Bid, Bax, p53 and Cytochrome C are involved in apoptosis, but XIAP (X-linked inhibitor of apoptosis protein) is the most significant cell death inhibitor that promotes resistance to chemotherapy, anti-cancer immune response and radiation. Hence, it is an important target for new anticancer agents [32]. Similarly, Survivin is a rare protein that is expressed both in normal as well as cancer cells, is mainly involved in several regulatory pathways required for tumor maintenance and is detected early in all cancer cells. These qualities of survival make it an attractive target for cancer therapy [33]. PARP (poly-ADP ribose polymerase) is involved in cell repair. Therefore, our study reports pro-apoptotic and anti-apoptotic protein gene expression after treatment with herbal melanin and revealed the downregulation of Bcl-2, Bcl-xl, XIAP and Survivin protein gene expression and that the Bid, Bax, p53, Cytochrome C and PARP protein genes were upregulated when treated with two different concentrations (80 µg and 160 µg) of herbal melanin. Our report proved that herbal melanin anticancer activity promoted apoptosis. Similarly, herbal melanin derived from N. sativa was evaluated for its anticancer activity against colorectal cancer and its effect on the mechanism of action for adenocarcinoma and metastatic colorectal cancer (mCRC) cell death and it was found that the activity was achieved via proliferation inhibition, enhanced ROS production, inhibition of Bcl-2 family proteins, release of cytochrome C and activation of caspase 3/7 [34]. Overall, herbal melanin inhibits the Bcl-2 family proteins and their genes, which are involved in apoptosis. The findings of this study open new avenues for further research on the molecular mechanisms underlying the influence of herbal melanin on cell proliferation and anti-apoptotic pathways. The potential of herbal melanin as a complementary or standalone therapeutic agent warrants extensive investigation, including clinical trials to validate its efficacy and safety in human subjects.

## 5. Conclusions

The present study concludes that herbal melanin was investigated for its anticancer activity against human breast adenocarcinoma (MDA-MB-231), human colorectal (HCT 116) cells proliferation as well as pro-apoptotic and anti-apoptotic proteins. The multifaceted impact of herbal melanin on cancer cells, as revealed in this study, adds a layer of depth to its potential therapeutic applications. The observed effective inhibition of cell proliferation in both MDA-MB-231 and HCT-116 cell types reinforces the promising nature of herbal melanin as a potent anticancer agent.

The downregulation of Bcl-2 family proteins, coupled with the upregulation of the pro-apoptotic protein p53, signifies a shift in the cellular environment towards promoting apoptosis—a crucial mechanism for eliminating aberrant cells. The activation of caspase 3 and 9 further corroborates the induction of apoptotic pathways, highlighting the specificity of herbal melanin in targeting key players in cell survival and death.

The proposition of herbal melanin as an alternative drug for the treatment of human breast adenocarcinoma and human colorectal cancers is an exciting prospect. The demonstrated efficacy, coupled with a better understanding of its mechanism of action, positions herbal melanin as a compelling candidate for further preclinical and clinical investigations. The comprehensive exploration of herbal melanin’s anticancer activity, along with the elucidation of its underlying mechanism of action, sets the stage for its potential role as an alternative drug in the treatment of human breast adenocarcinoma and colorectal cancers. 

## Figures and Tables

**Table 1:** Sequences of primers used for reverse transcription-quantitative polymerase chain reaction and antibodies.

Target	Primer Sequences	Primary Antibody
genes		(dilution 1:1000)
Bcl-2	F: 5′-AAGATTGATGGGATCGTTGC-3′	Bcl-2 (cat. no. sc-509)
	R: 5′-GCGGAACACTTGATTCTGGT-3′	
Bcl-xl	F: 5′-CCCAGAAAGGATACAGCTGG-3′	Bcl-xl (cat. no. sc-8392)
	R: 5′-GCGATCCGACTCACCAATAC-3′	
XIAP	F: 5′-GACAGTATGCAAGATGACGTCAAGTCA-3′	p53 (cat. no. sc-47698)
	R: 5′-GCAAAGCTTCTCCTCTTGCAG-3′	
Survivin	F: 5′-ACCGCATCTCTACATTCAAG-3′	β-Actin (cat. no. sc-47778)
	R: 5′-CAAGTCTGGCTCGTTCTC-3′	
Bid	F: 5′-CCTTGCTCCGTGATGTCTTTC-3′	Secondary Antibodies
	R: 5′-GTAGGTGCGTAGGTTCTGGT-3′	(dilution 1:10,000)
Bax	F: 5′-GTGCACCAAGGTGCCGGAAC-3′	Mouse anti-rabbit IgG-HRP (cat. no. sc-2357)
	R: 5′-TCAGCCCATCTTCTTCCAGA--3′	
Cytochrome c	F: 5′-GGCTGCAGTGTAGCTGTGAT-3′	(All Primary and secondary antibodies are purchased from Santa Cruz Biotechnology, Inc., Dallas, TX, USA.)
	R: 5′-GATGGAGTTTCCTTTATCTGTTGC-3′	
PARP	F: 5′-GGAAAGGGATCTACTTTGCCG-3′	
	R: 5′-TCGGGTCTCCCTGAGATGTG-3′	
P53	F: 5′-GAGGTTGGCTCTGACTGTACC-3′	
	R: 5′-TCCGTCCCAGTAGATTACCAC-3′	
Caspase 3	F: 5′-AGAACTGGACTGTGGCATTGAG-3′	
	R: 5′-GCTTGTCGGCATACTGTTTCAG-3′	
Caspase 9	F: 5′-TTCCCAGGTTTTGTTTCCTG-3′	
GAPDH	R: 5′-CCTTTCACCGAAACAGCATT-3′	
	F: 5′-AATCCCATCACCATCTTCCAG-3′	
	R: 5′-ATCAGCAGAGGGGGCAGAGA-3′	

Mean ± standard deviation (SD). Data points were collected for a minimum of three independent experiments. One-way ANOVA test was used to compare two groups and a value of *p* < 0.05 considered significant. The use of the mean ± standard deviation provides a clear representation of the data distribution. Additionally, conducting a minimum of three independent experiments added robustness to our findings, ensuring reliability. The choice of the one-way ANOVA test for group comparison is appropriate, and setting a significance level of *p* < 0.05 is a standard practice in scientific research. This statistical approach strengthens the validity of our results and contributes to the overall credibility.

## Data Availability

The data that support the findings of this study are available on request from the corresponding author.
